# Enhancement of CRISPR-Cas9 induced precise gene editing by targeting histone H2A-K15 ubiquitination

**DOI:** 10.1186/s12896-020-00650-x

**Published:** 2020-10-23

**Authors:** Sanum Bashir, Tu Dang, Jana Rossius, Johanna Wolf, Ralf Kühn

**Affiliations:** 1 Max-Delbrück-Centrum für Molekulare Medizin, 13125 Berlin, Germany; 2 Present Address: BioNTech Cell & Gene Therapies GmbH, Mainz, Germany; 3 Present Address: Glycotope GmbH, 13125 Berlin, Germany

**Keywords:** Precise gene editing, CRISPR, Cas9. Genome editing, Rad18, RNF169, BRCA1, tetR, Gal4, HR

## Abstract

**Background:**

Precise genetic modifications are preferred products of CRISPR-Cas9 mediated gene editing in mammalian cells but require the repair of induced double-strand breaks (DSB) through homology directed repair (HDR). Since HDR competes with the prevailing non-homologous end joining (NHEJ) pathway and depends on the presence of repair templates its efficiency is often limited and demands optimized methodology.

**Results:**

For the enhancement of HDR we redirect the DSB repair pathway choice by targeting the Ubiquitin mark for damaged chromatin at Histone H2A-K15. We used fusions of the Ubiquitin binding domain (UBD) of Rad18 or RNF169 with BRCA1 to promote HDR initiation and UBD fusions with DNA binding domains to attract donor templates and facilitate HDR processing. Using a traffic light reporter system in human HEK293 cells we found that the coexpression of both types of UBD fusion proteins promotes HDR, reduces NHEJ and shifts the HDR/NHEJ balance up to 6-fold. The HDR enhancing effect of UBD fusion proteins was confirmed at multiple endogenous loci.

**Conclusions:**

Our findings provide a novel efficient approach to promote precise gene editing in human cells.

**Supplementary information:**

**Supplementary information** accompanies this paper at 10.1186/s12896-020-00650-x.

## Background

**Fig. 1:**
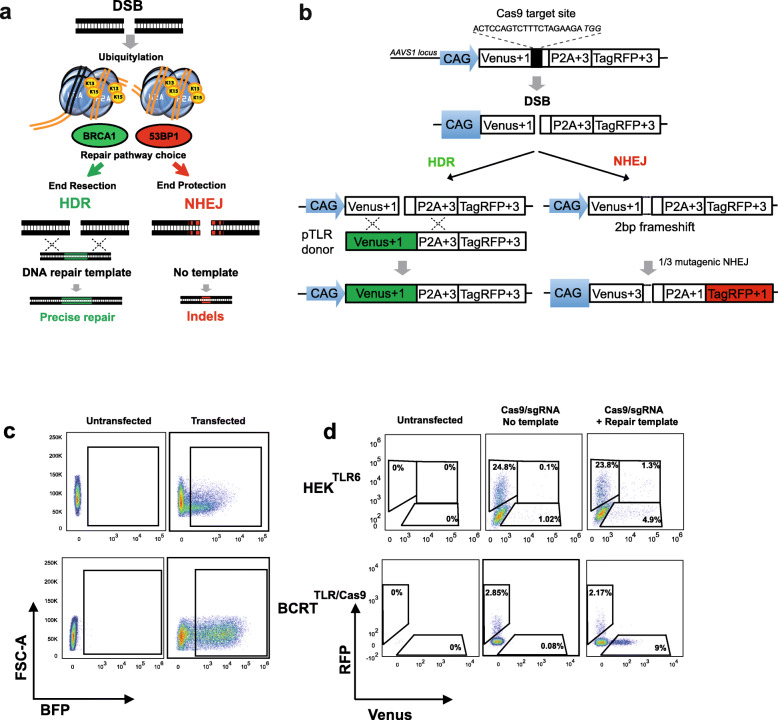
DSB repair assays in TLR reporter cells. **a** Diagram of DSB repair pathway choice and ubiquitination of histone H2A at DNA double-strand breaks (DSB). Upon DSB induction, regulatory proteins bind to ubiquitin at positions K13 and K15 via ubiquitin-binding domains (UBDs). The 53BP1 and BRCA1 regulatory proteins play an important role in DSB repair pathway choice. DSB repair is executed by repair proteins and leads either to non-homologous end ligation (NHEJ) or homology-directed repair (HDR). If the NHEJ pathway is chosen, the DNA ends religate, frequently associated with nucleotide deletions or insertions. The HDR pathway enables precise sequence modifications if a DNA repair template is available. **b** The ‘traffic light’ reporter (TLR) system indicates the ratio of DSB repair by NHEJ or HDR. Upon induction of DSBs in the target region using CRISPR-Cas9, RFP is expressed when repair by NHEJ results in deletions that shift translation into the RFP reading frame. Venus expression reports for HDR when an intact Venus coding region is cotransfected. **c** Gating scheme for BFP positive cells in transfected HEK and hiPS reporter cells. Single cells were gated by using a forward scatter (FSC-H vs. FSC-A) plot. Transfected cells were gated based on expression of BFP from transfected plasmids compared to non-transfected control. **d** TLR assay in HEK or hiPS reporter cells. At least 10,000 cells were analyzed per sample for the Venus or RFP positive population. Double positive cells in the HEK^TLR6^ clone are gated using an extra window

The RNA guided Cas9 nuclease is a versatile tool for genome editing in mammalian cells by creation of targeted double-strand breaks (DSBs) [1]. Gene editing at Cas9 induced DSBs is achieved by two alternative DSB repair pathways, either by non-homologous end joining (NHEJ) that leads to randomly sized small deletions or insertions (Indels), or by homology-directed repair (HDR) enabling precise sequence modifications that are copied from a repair template molecule. Since HDR is restricted to the S and G2 phases of the cell cycle [2] and requires the presence of a repair template it occurs notably less frequently than NHEJ, presenting a barrier for all applications that rely on precise sequence modifications, such as modelling of disease mutations or the correction of mutations in somatic gene therapy. To reinforce precise gene editing, tools or interventions are required that bias DSB repair pathway choice in favor of HDR and that promote HDR processing by the targeted delivery of DNA repair templates to DSBs. In particular the availability of repair templates may present a rate limiting factor for HDR. Previous approaches for the targeted delivery of repair templates used Cas9 fusion proteins with domains binding to a functional group that is incorporated into synthetic oligonucleotides or PCR fragments as donor templates and that are delivered into cells as combined Cas9-sgRNA-donor complexes [3–5]. However, it is presently unknown whether the link of the repair template molecule to Cas9 nuclease is the most effective way for codelivery, since the template is required during later steps of DSB repair. Previous approaches to promote DSB repair pathway choice in favor of HDR include the enrichment of cells in the S/G2 phase [6, 7], restriction of Cas9 activity to the S/G2 phase [8, 9], inhibition of NHEJ key molecules [10, 11] and the use of Cas9 fusion proteins with the HDR effector CtIP [12]. Nevertheless, these interventions do not directly target the protein complexes determining the repair pathway choice at the DSB ends, that presumably represent an effective target to promote HDR. The pathway choice for DSB repair is influenced by the interplay between the regulatory proteins BRCA1 and 53BP1, leading either to the resection or protection of DSB ends and the subsequent engagement of the HDR or NHEJ pathway (Fig. 1a) [13–15]. The 53BP1 protein has been identified as the key regulator for the initiation of DSB repair by NHEJ and represents a prime target for interventions aiming for suppression of NHEJ. 53BP1 is recruited to DSBs by recognition of the key Ubiquitin mark for damaged chromatin [16], set by the E3 Ubiquitin ligase RNF168 at Lysine 15 of histone H2A (H2A-K15^Ub^) in nucleosomes flanking the break sites (Fig. 1a) [17–20]. To suppress the recruitment of 53BP1 to DSBs, earlier studies used a dominant negative 53BP1 subdomain [21] or modified Rad18 [22] for masking of the H2A-K15^Ub^ site or developed the inhibitor i53, a mutant Ubiquitin that blocks the H2A-K15^Ub^ recognition domain of 53BP1 [23]. The inhibition of 53BP1 accumulation alleviates a barrier in the accessibility of HDR initiating factors at DSB sites and leads to a 2–3-fold stimulation of HDR. Furthermore, it has been found that H2A-K15^Ub^ together with the acidic patch on the nucleosome surface is also recognized by the Ubiquitin binding domains (UBD) of Rad18, RNF168 and RNF169 [24, 25]. Since the UBD of Rad18 and RNF169 bind H2A-K15^Ub^ with substantial higher affinity than RNF168 or 53BP1, the overexpression of Rad18 or RNF169 leads to the displacement of 53BP1 from DNA repair foci [26, 27]. In contrast to 53BP1, Rad18 and RNF169 also recognize the Ubiquitin mark at H2A-K13 but exhibit substantially lower affinity as compared to the H2A-K15^Ub^ site [25].

We reasoned that the UBD of Rad18 (Rad18^UBD^) and RNF169 (RNF169^UBD^) may provide powerful tools for the stimulation of HDR since they target high affinity H2A-K15^Ub^ binding sites at the hub of DSB repair. Hence, the fusion of Rad18^UBD^ and RNF169^UBD^ with proteins stimulating HDR could fulfill the dual purpose of loading DSB sites with effectors of choice and of NHEJ suppression through displacement of 53BP1. As effector domains either proteins that directly stimulate HDR can be used or fusions with DNA binding domains that link the DSB with a repair template that includes the respective binding site. Here, we present this novel approach for HDR stimulation by using a traffic-light DSB reporter (TLR) system for the quantitative detection of HDR and NHEJ events in human HEK and induced pluripotent stem cells. We found that the fusion of Rad18^UBD^ or RNF169^UBD^ with BRCA1 increases the ratio of HDR/NHEJ 3.6–4.1-fold. A comparable increase of HDR can be obtained by using UBD fusion proteins with the Tet repressor (TetR) or Gal4 DNA binding domains to enrich repair template molecules that include TetR or Gal4 binding sequences at DSB sites. Combined expression of a BRCA1-UBD together with a TetR- or Gal4-UBD is most effective, shifting in HEK293 cells the HDR/NHEJ ratio at the reporter and endogenous loci up to 6-fold.

## Results

### DSB repair assays in traffic light reporter cells

To quantitatively determine CRISPR/Cas9-induced DSB repair by HDR or NHEJ, we integrated a ‘traffic light’ reporter (TLR) construct into the Adeno-Associated Virus Integration Site 1 (AAVS1) locus of human HEK293 cells and human induced pluripotent stem cells (hIPSC). In HEK cells the reporter construct TLR-6 includes a CAG promoter for expression of a nonfunctional coding region for Yellow fluorescent (Venus) protein in reading frame + 1, disrupted by the replacement of codons 95–97 with a 23 bp gRNA target sequence from the mouse *Rosa26* locus (sgRosa), followed by a P2A peptide and the coding region for a red fluorescent (TagRFP) protein in reading frame + 2 (TLR-6) (Fig. 1b). CRISPR/Cas9-induced DSBs in the TLR-6 target region that are repaired via NHEJ and cause the deletion of 1 basepair (or of 1 + 3, 1 + 6 bp, etc.) shift the translation to the frame of P2A-TagRFP and are detectable in reporter cells by RFP expression. If an intact Venus coding region is provided as repair template and DSB repair occurs via the HDR pathway the reporter cells are detected by the expression of Venus (Fig. 1b). To generate a HEK293 reporter line, cells were transfected with an AAVS1 targeting vector carrying the TLR-6 insert along with Cas9 and an AAVS1-specific sgRNA expression plasmid. We selected a homozygously targeted HEK293 clone (HEK^TLR6^) for DSB repair assays. For activation of the reporter we transfected HEK^TLR6^ reporter cells with a vector for expression of Cas9, sgRosa and a blue fluorescent protein (BFP) together with a circular donor plasmid (pTLR-donor) for repair of the defective Venus reporter gene. The cells were analyzed 72 h after transfection by flow cytometry, gated on the BFP^+^ transfected population (Fig. 1c). Within the BFP^+^ population the frequency of Venus^+^ and of RFP^+^ cells was determined. Since HEK^TLR6^ cells are homozygous for the reporter construct a small population of double positive cells appears as well, undergoing HDR repair on one reporter allele and a mutagenic NHEJ event on the other reporter allele. The total number of Venus and RFP positive cells was calculated by addition of the values of single and double positive cells. As shown in Fig. 1d, using HEK^TLR6^ cells we observed 6.2% of Venus^+^ and 25.1% of RFP^+^ cells indicating HDR or RFP activating NHEJ repair events of the reporter in the ratio of 0.25, as compared to 24.9% RFP^+^ and background levels of 1.1% Venus^+^ cells in a control lacking pTLR-donor. The unexpected background of Venus^+^ cells in the absence of the repair template was explained by a specific 14 bp deletion event that occurs in a small fraction of cells and reconstitutes the Venus reading frame and the critical arginine codon 96 (Figure S1). Transfection samples including pTLR-donor were subtracted for background levels determined in the same experiment. In hiPSC we used a previously described TLR construct [11] that is similar to TLR-6 except that codons 117–152 of Venus were replaced by the sgRosa target sequence and the P2A-TagRFP is expressed in the + 3 reading frame upon the deletion of 2 basepairs (or of 2 + 3, 2 + 6 bp, etc.). In hiPSC we targeted one AAVS1 allele using a vector carrying the TLR insert and inserted a vector for the constitutive expression of Cas9 into the second AAVS1 allele. Upon transfection of hiPSC reporter cells with sgRosa and pTLR-donor plasmids we observed 2.17% of RFP^+^ and 9% of Venus^+^ cells (Fig. 1d), indicating that HDR repair in hiPS cells is more proficient than mutagenic NHEJ as compared to HEK cells. No RFP^+^ or Venus^+^ cells were observed in untransfected controls.

### DSB repair modification by Rad18^UBD^ and RNF169^UBD^ fusion proteins in traffic light reporter cells

**Fig. 2:**
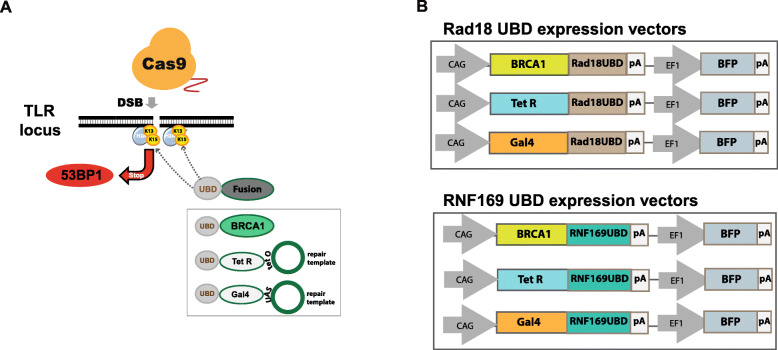
UBD expression vectors and transfection of TLR reporter cells. **a** UBD fusion proteins can compete with 53BP1 for binding at H2A-K15 and suppress NHEJ. BRCA1-UBD fusion proteins can direct this HDR factor to DSBs, while fusion with Tet repressor (TetR) or Gal4 attract the repair template molecule that include TetO or UAS binding sites, supporting HDR processing. **b** Plasmids constructed for expression of the Ubiquitin binding domain (UBD) of Rad18 of RNF169 in fusion with the coding region of BRCA1, Tet repressor or Gal4, driven by the CAG promoter. Plasmids include an EF1-BFP reporter gene to facilitate the FACS-based analysis

For the fusion of the UBD binding domains from RAD18 or RNF169 we used two types of proteins: either the endogenous BRCA1 protein acting as HDR enhancer or a DNA binding domain from bacterial Tet repressor (TetR) or yeast Gal4 for recognition of sequence motifs that must be included in the targeting vector. Upon DSB induction and H2A-K15 ubiquitination these fusion proteins should cover the DSB ends, compete with 53BP1, and support HDR by increasing the local concentrations of BRCA1 and the HDR repair template (Fig. 2a). For the expression of BRCA1, TetR or Gal4 fusions with the UBD of Rad18 (Rad18^UBD^) or RNF169 (RNF169^UBD^) we used plasmids carrying a CAG promoter and a BFP reporter gene (Fig. 2b). To enable the accumulation of repair templates at DSB sites by TetR- or Gal4-UBD proteins we cloned Tet operator (tetO) or UAS recognition sequences into the pTLR-donor vector adjacent to the TLR homology region.

**Fig. 3:**
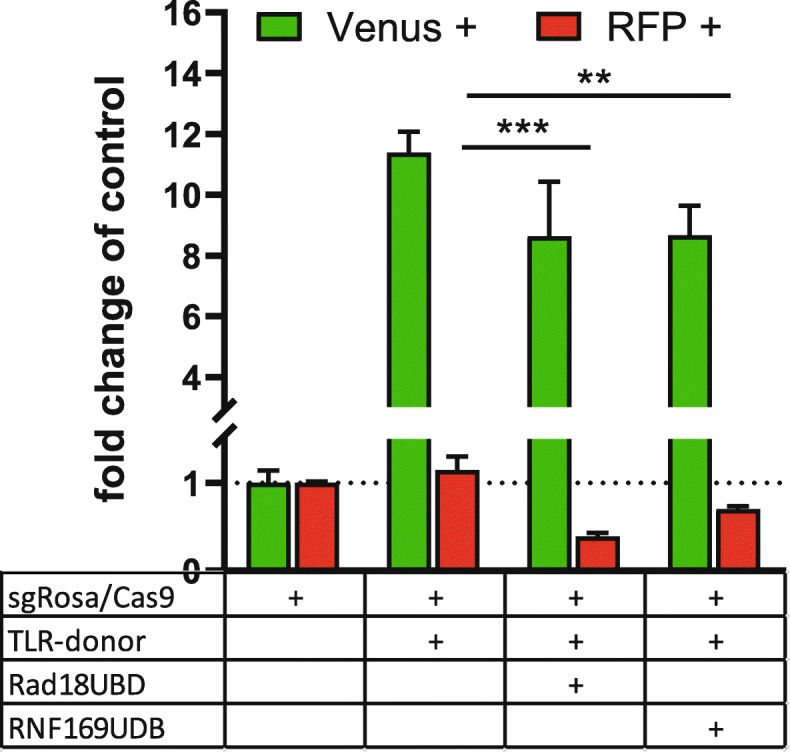
Expression of isolated UBDs in HEK^TLR6^ cells**.** HEK^TLR6^ cells were cotransfected in triplicates using sgRNA, Cas9, pTLR-donor and unfused UBDs of Rad18 and RNF169, driven by the CAG promoter. Transfected cells were gated based on expression of BFP and the percentage of Venus (HDR) (green bars) or RFP (NHEJ) (red bars) positive cells was determined by FACS analysis. The expression of these UBDs decreases NHEJ as detectable by RFP expression but do not enhance HDR. Gene editing efficiency is expressed as percentage of Venus (HDR) (green bars) or RFP (NHEJ) (red bars) positive cells normalized to values of sample 1 (sgRosa/Cas9). Data are shown as mean values ± SD from two independent experiments, each with three replicates per samples, normalized to the values of the first (control) sample. Significance of values in comparison to the control with sgRosa/Cas9 and TLR-donor was determined by two-way ANOVA and Dunnett’s multiple comparison tests with **P < 0.01, ***P < 0.001. Raw data are shown in the Supplementary data file

For DSB repair assays HEK^TLR6^ cells were cotransfected with plasmids for expression of Cas9, sgRosa, pTLR donor and plasmids for the expression of UBD fusion proteins. hiPS reporter cells exhibiting constitutive Cas9 expression were cotransfected with plasmids for sgRosa, UBD fusion proteins and pTLR-donor vector. Three days after transfection the frequency of Venus^+^ and RFP^+^ cells was determined by FACS. The ratio of Venus^+^/RFP^+^ cells is used as indicator for DSB repair choice by HDR or mutagenic NHEJ leading to RFP expression as determined by FACS analysis. We first expressed the Rad18^UBD^ or RNF169^UBD^ domain alone to assess their effect in HEK^TLR6^ reporter cells. As shown in Fig. 3 the expression of Rad18^UBD^ slightly diminished the Venus^+^ cell population but significantly reduced the number of RFP^+^ cells by 60%. A similar, but weaker effect was observed for RNF169^UBD^ that reduced the frequency of RFP^+^ cells by 30% (Fig. 3). Thus, the expression of Rad18^UBD^ and RNF169^UBD^ domains alone inhibits NHEJ but does not enhance HDR. The reduction of NHEJ repair is in agreement with previous findings that Rad18^UBD^ and RNF169^UBD^ domains compete with 53BP1 for H2A-K15^Ub^ binding sites [26, 27]. We confirmed in our experimental settings that the Rad18^UBD^ domain colocalizes with phosphorylated H2AX at DSB repair foci (Figure S2).

**Fig. 4:**
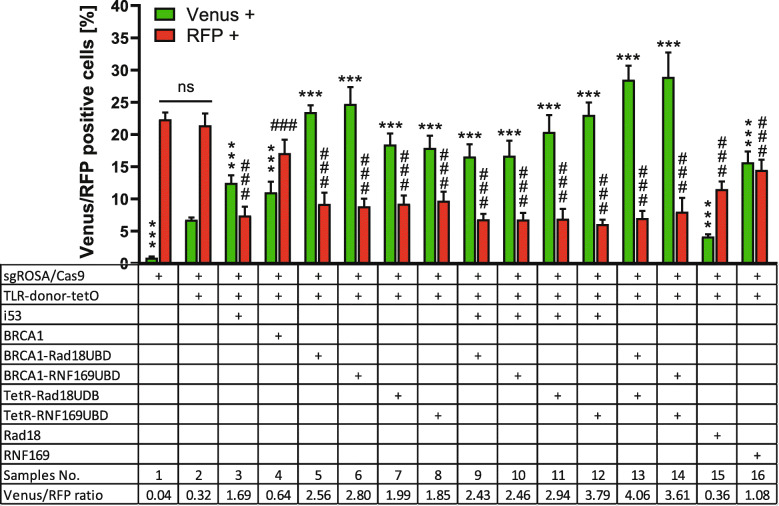
Fluorescence-based DSB repair assay using Rad18^UBD^ and RNF169^UBD^ fusion proteins in HEK^TLR6^ reporter cells**.** Fusion constructs for BRCA1 or TetR with Rad18^UBD^ or RNF169 ^UBD^ were cotransfected with the TLR HDR repair template (TLR-donor-tetO), sgRNA and Cas9 into HEK^TLR6^ cells. The frequency of Venus^+^ cells (green bars) and RFP^+^ cells (red bars) within the population of BFP^+^ cells was measured by FACS analysis 72 h after transfection and used to calculate the ratio of Venus/RFP positive cells. The X-axis shows the transfected samples and the selection of cotransfected plasmids below. Samples 1 and 2 are controls showing the basic frequency of Venus^+^ and RFP^+^ cells upon transfection with Cas9 and sgRNA or in combination with TLR-donor-tetO as repair template. Data from four independent experiments, each with three replicates per sample, are represented as mean values ± SD. Statistical significance of samples 3–16 in comparison to the control sample 2 was determined by two-way ANOVA and Dunnett’s multiple comparison tests with **P < 0.01, ***P < 0.001 (HDR) and ##P < 0.01, ###P < 0.001 (NHEJ). Raw data are shown in the Supplementary data file

Next, we assessed the effect of UBD fusion proteins on HDR and NHEJ events in HEK^TLR6^ reporter cells by FACS analysis (Fig. 4). First we determined the effect of BRCA1 alone and of BRCA1 in fusion with UBDs of Rad18 or RNF169 in comparison to i53 as NHEJ inhibitor. The expression of i53 increased the Venus/RFP ratio from 0.32 to 1.69 (Fig. 4 sample 3) as compared to the control (sample 2). The expression of BRCA1 (sample 4) lead only to a smaller reduction of RFP^+^ cells and an increase of Venus^+^ cells (Venus/RFP ratio: 0.64). In contrast, the expression of BRCA1-Rad18^UBD^ or BRCA1-RNF169^UBD^ fusion proteins (samples 5 and 6) leads to a more than 3-fold increase of Venus^+^ cells and to the reduction of RFP^+^ cells by more than half, shifting the Venus/RFP ratio to 2.56 and 2.8, respectively. The expression of TetR-Rad18^UBD^ or TetR-RNF169^UBD^ fusion proteins (samples 7 and 8) lead to a more than 2-fold reduction of RFP^+^ cells and more than 2-fold increase of Venus^+^ cells, shifting the Venus/RFP ratio to 1.99 and 1.85, respectively. The combination of i53 with BRCA1-Rad18^UBD^ or BRCA1-RNF169^UBD^ (samples 9 and 10) did not lead to a further increase of the Venus/RFP ratio as compared to the use of the fusion proteins alone. In contrast, the effect of TetR-Rad18^UBD^ and TetR-RNF169^UBD^ (samples 11 and 12) was enhanced in the presence of i53, shifting the Venus/RFP ratio to 2.94 and 3.79, respectively. Next, we explored whether BRCA1- and TetR-Rad18^UBD^ or -RNF169^UBD^ fusion proteins can be combined to further increase HDR frequency. As compared to the use of single fusion proteins, we observed in both combinations (samples 13 and 14) an increase of the Venus/RFP ratio to values of 4.06 or 3.61, respectively. Finally, we compared the effect of UBD fusions with the full length Rad18 or RNF169 proteins (samples 15 and 16). Expression of Rad18 leads to a Venus/RFP ratio of 0.36, comparable to the control, whereas RNF169 expression moderately increased the Venus/RFP ratio to a value of 1.08.

In addition, we tested the DNA binding domain of the yeast transcription factor Gal4 as alternative to TetR and included ten copies of its 17 bp UAS recognition sequence into pTLR-donor (pTLR-donor-UAS). The expression of Gal4-Rad18^UBD^ or Gal4-RNF169^UBD^ alone increased the Venus/RFP ratio in HEK^TLR6^ cells from 0.34 to values of 1.37 or 2.0 and to 3.14 or 3.29 together with BRCA1 fusion proteins (Figure S3), similar to the effect of TetR-UBD fusion proteins.

For the sequence-based analysis of gene editing products we amplified the reporter target region using cells from one of the HEK^TLR6^ FACS assay (Fig. 4) and performed amplicon sequencing. The NGS based analysis of repair products showed essentially the same effects as observed in the FACS-based analysis. The transfection of Cas9, sgRosa and pTLR-donor-tetO resulted in a HDR/NHEJ ratio of 0.42 that increased up to 2.22 or 2.52 by the coexpression of BRCA1- and TetR-Rad18^UBD^ or BRCA1- and TetR-RNF169^UBD^ fusion proteins, respectively (Figure S4). As expected for sequence analysis the fractions of NHEJ products were higher and the fraction of HDR products lowered, since FACS analysis detects only Indel products that lead to RFP expression in reading frame + 2. To this end, we analyzed the distribution of reading frames and found that NHEJ repair products in frame + 2 were prevailing in all samples (Figure S5). Therefore, RFP expression measured by FACS is able to detect the majority of NHEJ repair products and the coexpression of DSB repair modifiers did not bias the distribution of reading frames among the Indel products.

We further assessed the activity of Rad18^UBD^ and RNF169^UBD^ fusion proteins in hiPS TLR reporter cells by FACS. In hIPC cells the expression of i53, BRCA1 or BRCA1-Rad18^UBD^ proteins did not lead to a significant increase of Venus^+^ cells, in contrast to TetR-UBD fusions. BRCA1 and TetR fusion proteins in combination lead to a 4–6-fold increase of the Venus/RFP ratio (Figure S6, samples 12, 13) as compared to the control (sample 2), similar to the observation in HEK^TLR6^ cells (Fig. 4). The expression of full length Rad18 or RNF169 proteins had no significant effect on the number of Venus^+^ cells.

These results show that the UBD of Rad18 and RNF169 are effective tools for the manipulation of DSB repair pathway choice at the reporter locus. While the UBD alone reduces NHEJ, its fusion with BRCA1 or with a DNA binding domain associating with the repair template increases DSB repair by HDR. Each of the fusion proteins alone exhibits an effect on the HDR/NHEJ ratio that is stronger than the i53 NHEJ inhibitor. Highest levels are reached by the combination of two Rad18^UBD^ or RNF169^UBD^ fusion proteins. Under these conditions, HDR events in HEK^TLR6^ cells are increased > 2-fold and NHEJ events reduced > 2-fold, shifting the HDR/NHEJ ratio by a factor of up to 6-fold (Figure S4 sample 2 vs 14).

### Targeting of endogenous genes in HEK cells

**Fig. 5:**
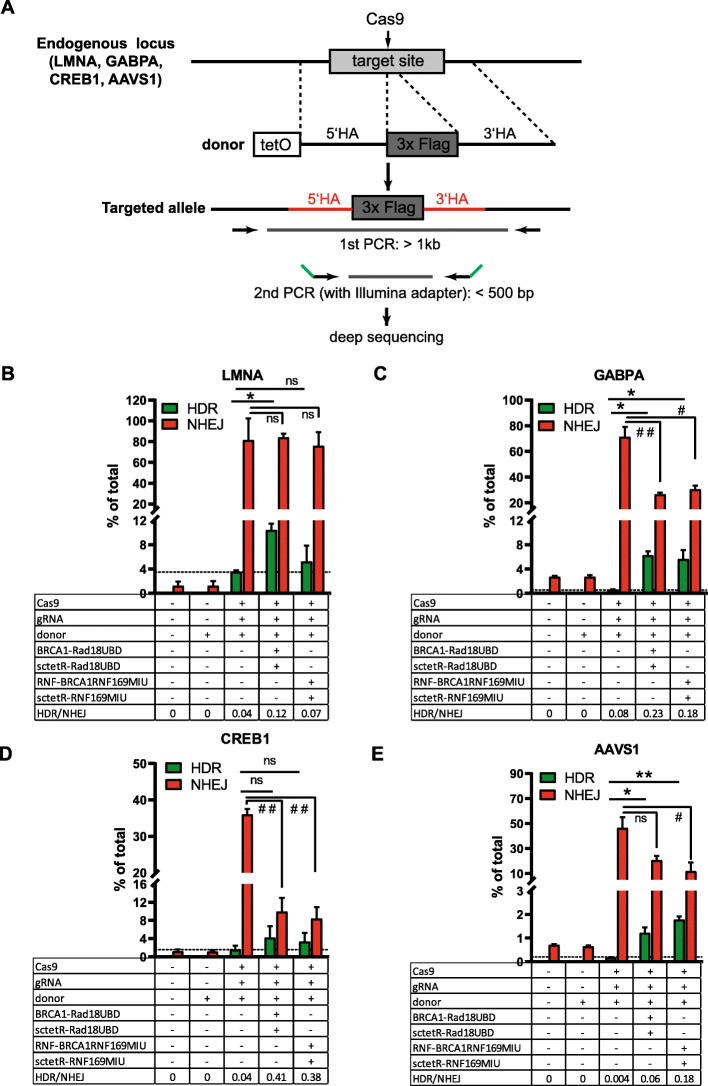
Knockin of a triple FLAG Tag into endogenous genes in HEK cells. **a** Schematic drawing of the targeting strategy at the endogenous LMNA, GABPA, CREB1, and AAVS1 loci. HEK cells were transfected with expression vectors for Cas9, sgRNA and a donor vector with tetO elements for introduction of a triple FLAG sequence into the first or last exon of the GABPA (exon 10), CREB1 (exon 9) or LMNA (exon 1) gene and into the AAVS1 site of the PPP1RC12C (first intron) gene, respectively. Three days after transfection, genomic DNA was isolated and the target region was amplified by a two-step PCR reaction using the indicated primers (arrows, green: Illumina adapter). The secondary PCR products were sequenced by amplicon sequencing. **b**, **c**, **d**, **e**. DSB repair events were quantified by deep sequencing reads for each target gene. The fraction of reads showing HDR (green bars) or Indel events (red bars) is shown on the Y-axis in relation to the total number of reads showing wildtype or gene editing events and was used to calculate the ratio of HDR/NHEJ DSB repair. The table shows the selection of cotransfected plasmids of each sample for the expression of Cas9, sgRNA and BRCA1- and TetR- with Rad18^UBD^ or RNF169^UBD^ fusion proteins. Data are presented as mean values ± SD from two independent experiments. *P < 0.05, **P < 0.01 (HDR) and #P < 0.05, ##P < 0.01 (NHEJ); t-test. Raw data are shown in the Supplementary data file

We further confirmed the effect of UBD fusion proteins on DSB repair by targeting of five endogenous loci in the HEK cell genome. The LMNA (Lamin A), GABPA (GA binding protein transcription factor alpha subunit), CREB1 (cyclic AMP-responsive element-binding protein 1), and the AAVS1 loci were targeted for the insertion of a 78 bp sequence encoding a triple FLAG Tag. Each gene was targeted using a repair template vector with tetO elements and homology regions (0.5–0.9 kb) flanking the Tag insertion (Fig. 5a). Upon the transfection of HEK cells with donor vector together with Cas9, sgRNA and UBD fusion proteins the target regions were amplified and gene editing events were analysed by amplicon sequencing (Fig. 5a). This analysis revealed for LMNA, GABPA and AAVS1 but not CREB1 a significant increase of HDR upon coexpression of BRCA1- and TetR-Rad18^UBD^ fusion proteins as compared to the control transfected only with Cas9, sgRNA and donor template (Fig. 5b-d). At CREB1, GABPA and AAVS1 but not LMNA, the coexpression of Rad18^UBD^ fusion proteins also lead to a significant decrease in NHEJ events. At the GABPA and AAVS1 loci the decrease of NHEJ was comparable to CREB1, but the increase in HDR was considerably stronger upon coexpression of Rad18^UBD^ fusion proteins, notably 11.2-fold for GABPA and 7.1-fold for AAVS1 (Fig. 5c, e). This shifted the HDR/NHEJ balance of DSB repair at the GABPA and AAVS1 loci by a factor of 29 and 15, respectively. Likewise the coexpression of BRCA1- and TetR-RNF169^UBD^ fusion proteins shifted the HDR/NHEJ ratio at all indicated loci by a factor of at least 1.8 (Fig. 5b-e). Their combined expression lead to a 1.5, 10, 2.1 and 10.5-fold increase in HDR for the LMNA, GABPA, CREB1, and AAVS1 loci respectively, whereas NHEJ was suppressed at least 2.3 fold at the three latter loci (Fig. 5b-e).

**Fig. 6:**
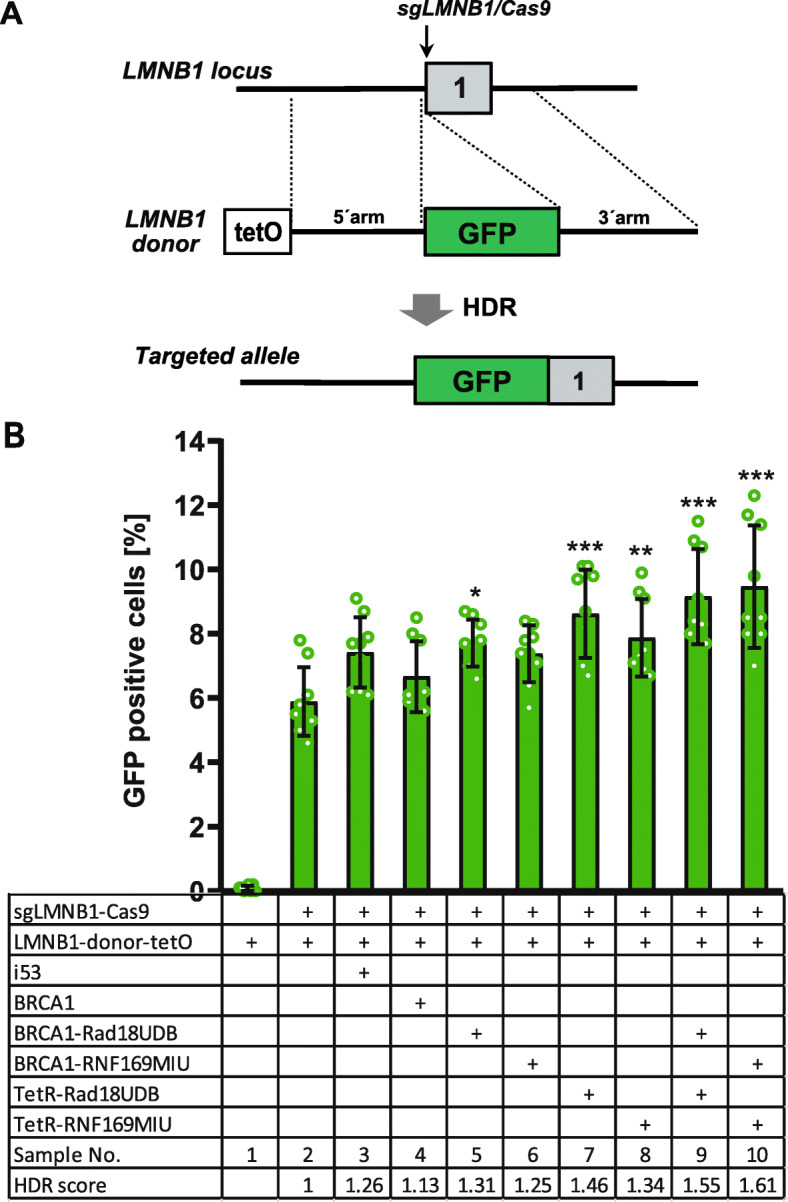
Knockin of GFP into the LMNB1 gene. **a** Schema of the human LMNB1 gene exon 1, Cas9 target site and the donor vector for the insertion of the GFP coding region downstream of the LMNB1 start codon, flanked by 5′- and 3′- homology arms. Upon homologous recombination (HDR) a GFP/LMNB1 fusion protein is produced. **b** HEK cells were transfected either with the tetO modified LMNB1 targeting vector (LMNB1-donor-tetO) alone or together with an expression vector for LMNB1-sgRNA and Cas9 (sgLMNB1/Cas9) or with expression vectors for BRCA1, BRCA1-Rad18^UBD^, BRCA-RNF169^UBD^, TetR-Rad18^UBD^ or TetR-RNF169^UBD^ as shown in the table. The HDR frequency was determined as the number of GFP positive cells using FACS analysis 10 days after transfection and its relative increase in comparison to the control sample 2 is given as HDR score. Statistical significance of samples 3–10 in comparison to the control sample 2 was determined by ordinary one-way ANOVA and Dunnett’s multiple comparison tests with *P < 0.05, **P < 0.01, ***P < 0.001. Data from three independent experiments, each with three replicates per sample, are presented as mean values ± S.D. Raw data are shown in the Supplementary data file

For targeting of the LMNB1 (Lamin B1) gene we used a donor vector with tetO elements and a larger insert for the N-terminal fusion of GFP with LMNB1 (Fig. 6a). HEK cells were transfected either with Cas9, sgRNA and the LMNB1 donor alone or together with UBD fusion proteins and after 10 days the HDR frequency was determined by FACS as the number of GFP^+^ cells. As compared to the control (Fig. 6b, sample 2) the coexpression of a single Rad18^UBD^ or RNF169^UBD^ fusion protein increased the number of GFP^+^ cells by up to 32% (sample 5–8). The coexpression of TetR fusions (sample 7 and 8) or both types of Rad18^UBD^ (sample 9) or RNF169^UBD^ fusion proteins (sample 10) significantly increased the number of GFP^+^ cells by up to 49%. Taken together, these results demonstrate that Rad18^UBD^ and RNF169^UBD^ fusion proteins also support HDR at endogenous target genes in HEK cells. The strongest effects were observed by the combined expression of UBD fusion proteins with BRCA1 and TetR together with repair donor vectors that include tetO binding sites.

## Discussion

We show that the H2A-K15 Ubiquitin mark at CRISPR-Cas9 induced DSBs can be utilized for shifting DSB repair towards HDR, supporting precise gene editing. This is achieved by using the Ubiquitin binding domain of Rad18 or RNF169 in fusion with BRCA1 as HDR enhancer or the TetR or Gal4 DNA binding domain for connecting the repair vector to the DSB site. Since HDR requires the appropriate pathway choice and the presence of the repair template we achieved best results by the combined expression of both types of fusion proteins. These combinations strongly increased the ratio of HDR/NHEJ repair at multiple genomic targets.

Previous studies for HDR stimulation did not actively target DNA damage ubiquitination but either used Cas9 fusion proteins [4, 5, 12], targeted enzymes at earlier or later stages of DSB repair [10, 11, 28], confined Cas9 activity to the S/G2 cell cycle phases [6–9] or suppressed NHEJ by interfering with 53BP1 binding to H2A-K15ub using i53 [23], a dominant-negative 53BP1 subdomain [21] or a modified Rad18 protein [22]. In contrast, our UBD fusion protein approach manipulates DSB repair pathway choice not only by targeting of the 53BP1 binding site but also enables the active positioning of HDR enhancers and donor vectors at DSB sites. The former but not the latter feature can be also reached by small molecule (SCR7) [11] or peptide (i53) [23] inhibitors of NHEJ that are conveniently used in tissue culture. However, our direct comparison of UBD fusion proteins to the peptide inhibitor of 53BP1 showed that each fusion protein exhibits a stronger effect than i53. Highest levels (up to 6-fold in HEK^TLR6^ cells) were reached by the combination of two Rad18^UBD^ or RNF169^UBD^ fusion proteins.

The initial configuration of this approach as presented here is using the full length BRCA1 protein as HDR enhancer and a second UBD fusion protein with a TetR or Gal4 DNA binding domain for the attraction of the donor vector. To avoid the expression of two modifier proteins in future further studies will be required to define whether its design can be simplified by use of BRCA1 subdomains and whether the HDR enhancing domain can be combined with a DNA binding domain into a single fusion protein. Furthermore, it will be interesting to test whether BRCA1 can be replaced by one of its interaction partners, such as BRCA2 or Palb2. In addition, the DNA binding domains derived from TetR and Gal4 may be replaced by other domains for the recognition of double-stranded or single-stranded DNA donor templates, as shown for the link between Biotin or Benzylguanine conjugated oligodeoxynucleotides and Cas9 fusions with the SNAP-Tag or Avidin [4, 5]. Such modifications could result into a streamlined system for delivery into cells or tissues as plasmid, mRNA or as recombinant HDR enhancer protein together with Cas9/gRNA complexes by microinjection, electroporation or as nanoparticles.

In this study we employed a traffic light reporter system for the assessment of DSB repair activity by HDR and NHEJ in HEK and hiPS cells that we used earlier to validate DNA Ligase IV as target for NHEJ suppression. Since the TLR-6 reporter enables to detect only a fraction of NHEJ events by RFP expression upon the reconstitution of the + 2 reading frame and it was recently shown that the spectrum of DSB repair products is biased depending on the target sequence [29, 30], we compared the analysis of HEK^TLR6^ reporter cells by FACS and amplicon sequencing. These results showed that for the TLR-6 reporter DSB repair products in the reading frame + 2 were dominating and its RFP expression reflects the majority of mutagenic NHEJ events. Nevertheless, due to the lack of reporter activity in the other reading frames the FACS based analysis leads to an overestimation of the ratio of DSB repair by HDR or NHEJ as compared to amplicon sequencing. Based on the sequencing results the HDR/NHEJ balance at the reporter locus is shifted up to 6-fold in the presence of BRCA1 and TetR fusion proteins. A similar strong shift was observed at the GABPA, CREB1 and AAVS1 loci whereas at LMNA the suppression of NHEJ was not pronounced, pointing to a locus variability that requires further investigation. Furthermore, it is possible that the positive effect of UBD fusion proteins will be greatest if highly specific sgRNAs are used together with an off-target improved Cas9 nuclease, since the presence of additional DSBs at off-target sites may divert UBD fusion proteins from acting at the on-target site. To this end it will be interesting to compare the effect of using sgRNAs together with wildtype Cas9 in comparison to the off-target improved eCas9 [31], Cas9-Hifi [32] and other variants. In the present work we did not discriminate between DSB repair choice in the G1 and S/G2 cell cycle phases. We anticipate that HDR enhancement occurs primarily in the S/G2 phases in which the HDR pathway is fully available. It has been shown that HDR can be at least partially reactivated in G1 by the combined suppression of 53BP1 and the expression of a degradation resistant Palb2 mutant together with a phosphomimetic CtIP mutant [33]. Therefore it will be interesting to determine the effect of the H2A-K15^Ub^ approach specifically on DSB repair in the G1 phase and whether its current configuration or further modifications will enable HDR mediated DSB repair in the G1 phase or in resting cells. Based on previous studies [24, 27] we assume that the expression of Rad18^UBD^ or RNF169^UBD^ fusion proteins leads to the partial displacement of 53BP1 from its ubiquitin binding sites at DSBs. In the present work we did not study competition with 53BP1 but confirmed the presence of Rad18UBD protein in γH2AX foci.

In the current format of using plasmid based expression vectors we expect that the H2A-K15^Ub^ approach can readily support applications of precise gene editing in human cell lines such as the modelling or correction of disease causing mutations by cotransfection with Cas9/sgRNA vectors. In the format of recombinant proteins its applications may be extended in future to primary human cells such as hematopoietic stem cells, muscle satellite or other primary cells to assess its utility for the precise correction of mutations required for somatic gene therapy. Since DSB repair mechanism are conserved in evolution and DSB associated 53BP1 foci have been identified in various mammals, Xenopus and zebrafish [34–37], we envision that the targeting of 53BP1 binding sites can also be applied for HDR enhancement in other vertebrate species.

## Conclusions

The present study describes a new strategy for the enhancement of HDR in human cells. DSB repair pathway choice can be redirected by fusion proteins of Ubiquitin binding domains that localize at DSB sites, shifting the HDR/NHEJ balance several fold. These findings provide an efficient approach to promote precise gene editing in human cells, supporting its applications for research and therapy.

## Methods

### Plasmid constructions

pAAVS1-TLR6 was constructed by generation of a 1552 bp fusion PCR product from a 329 bp PCR fragment (using primers TLRvenus-1 and TLR6–1) and a 1247 bp PCR fragment (using primers TLR6–2 and TagRFP-3), using pAAVS1-TLR donor (Addgene ID 64215) [11] as template, into the backbone of plasmid pCAG-venusTarget+1P2A + 3TagRFP (opened with PacI and MluI), resulting into pCAG-TLR6. pCAG-TLR6 was used for isolation of a 3.2 kb AscI-AsiSI fragment that was ligated into pAAVS1-TLR (opened with AsiSI and AscI), resulting into pAAVS1-TLR6, serving as AAVS1 targeting vector with the TLR6 reporter insert. pU6Rosa-CAG-Cas9 for expression of Rosa26 sgRNA and *Streptococcus pyogenes* (Sp) Cas9 was constructed by ligation of the DNA oligonucleotides sgRosa-A and sgRosa-B into the BbsI sites downstream of a human U6 promoter into plasmid pU6(BbsI). The U6-sgRosa cassette was recovered as AscI fragment and inserted into pCAG-Cas9-bpA-EF1-BFP, upstream of the CAG promoter driving SpCas9 expression, followed by a BFP coding region under control of the human EF1α promoter. Plasmid pTLR-donor was generated by whole plasmid PCR amplification using 5′-phosphorylated primers TLRtv-1 and TLRtv-2 and pTLR-repair (Addgene ID 64322) [11] as template, followed by DpnI digestion of the template and religation of the PCR fragment. The modification of pTLR-repair removes the Start codon of the Venus coding region, eliminating background fluorescence upon transient transfection. For construction of pTLR-donor-tetO, pTLR-donor was opened with SalI and ligated with a 448 bp SalI fragment from plasmid pTREtightbi, a derivative of pTREtight (Clontech) that contains seven Tet operator elements. For construction of plasmid pTLR-donor-UAS, pTLR-donor was opened with SalI and ligated with two tandem copies of a 151 bp PCR fragment amplified with primers UAS-for/UAS-rev, each containing 5 UAS elements, using plasmid pUAS-luc2 (Addgene ID 24343) as template. Plasmid pCAG-Rad18UBD was constructed by generation of a 225 bp PCR fragment, including the UBD domain of human Rad18 between codon 191–240, amplified from plasmid myc-hRad18 (Addgene ID 68827) as template using primers Rad18-for and -rev. The PCR product was incubated with DpnI to remove the plasmid template, digested with PacI and MluI and ligated into the backbone of plasmid pCAG-Rad51-bpA-EF1-BFP opened with Pac and MluI. Plasmid pU6Rosa-CAG-Rad18 for expression of Rad18 was constructed by amplification of a 1536 bp PCR product, including the full length human Rad18 coding region, using primers Rad18-for2 and -rev2 and Addgene plasmid 68,827 as template. The PCR product was incubated with DpnI, digested with PacI and MluI and ligated into the PacI-MluI sites of plasmid pU6Rosa-CAG-Rad51-bpA-EF1BFP, replacing the Rad51 coding region. Plasmid pCAG-RNF169UBD for expression of the RNF169UBD was constructed by amplification of a 219 bp PCR fragment, including the UBD domain of human RNF169 between codon 662–709, using plasmid pcDNA5-FRT/TO-Flag-RNF169 (Addgene ID 74243) as template with primers RNF169-for2 and -rev. The PCR product was incubated with DpnI, digested with PacI and MluI and ligated into the backbone of plasmid pCAG-Rad51-bpA-EF1-BFP opened with Pac and MluI. Plasmid pCAG-RNF169 for expression of the full length human RNF169 was constructed by ligation of a 2167 bp PCR product amplified with primers RNF169-for and –rev (using a synthetic gene as template) into the backbone of plasmid pCAG-Rad51-bpA-EF1-BFP opened with Pac and MluI. Plasmid pCAG-BRCA1 for expression of BRCA1 was constructed by amplification of 5640 bp PCR fragment, including the full length human BRCA1 coding region, using plasmid pDEST-FRT/T0-GFP-BRCA1 (Addgene ID 71116) as template and primers BRCA1-for and -rev. The PCR product was incubated with DpnI, digested with PacI and MluI and ligated into the backbone of plasmid pCAG-Rad51-bpA-EF1-BFP opened with Pac and MluI. Plasmid pCAG-i53 was constructed by amplification of a 273 bp PCR fragment, including the i53 coding region, using plasmid pcDNA3-Flag-UbvG08 (Addgene ID 74939) as template and primers i51-for and -rev. The PCR product was incubated with DpnI, digested with PacI and MluI and ligated into the backbone of plasmid pCAG-Rad51-bpA-EF1-BFP opened with Pac and MluI. Plasmids pCAG-BRCA1-, pCAG-TetR-, and pCAG-Gal4-Rad18UBD for expression of Rad18UBD fusion proteins were constructed by amplification of specific PCR products that were digested with PacI and PvuI and ligated into the PacI site of pCAG-Rad18^UBD^. BRCA1 was amplified as 5681 bp PCR product using primers BRCA1-for and -rev2 and pCAG-BRCA1 as template, The TetR coding region was amplified as 1445 bp PCR fragment using primers TetRsc4-for and –rev and plasmid pU6MS2Rosa-CAG-MS2ditetRsc as template. pU6MS2Rosa-CAG-MS2ditetRsc was generated by cloning of a 739 bp PCR fragment amplified with primers scTetR-for2 and rev2 from pU6MS2Rosa-CAG-MS2ditetR into the PmlI site of pU6MS2Rosa-CAG-MS2ditetR to duplicate the TetR coding region, separated by 5 copies of a (GGGGS) linker, to generate a single chain Tet repressor coding region as described. We used a single chain TetR that combines two TetR monomers separated by a linker sequence [38] to enable the binding of TetR fusion proteins as a single protein. The Gal4 DNA binding domain was amplified as 561 bp PCR fragment from pActPL-Gal4DBD (Addgene ID 15304) using primers Gal4-for and –rev. The same PCR products were ligated into the PacI site of pCAG-RNF169UBD to generate the plasmids pCAG-BRCA1-RNF169UBD, pCAG-TetR-RNF169UBD and pCAG-Gal4-RNF169UBD for the expression of RNF169UBD fusion proteins. The AAVS1 targeting vector for Cas9 expression pAAVSI-NEOwt-CAG-Cas9v3a-bpA was generated by isolation of a 4.7 kb PacI-XhoI fragment isolated from pCAG-Cas9v3a-bpA followed by ligation into the AflII and SalI site of pAAVS1-NEOwt CAG-tetR-donor U6 acceptor, a derivative of Addgene plasmid 60,431. PX458_GABPA_2 and PX458_CREB1_1 were a gift from Eric Mendenhall & Richard M. Myers (Addgene plasmid # 64255 and 64,939). For cloning of pU6LMNB1-CAG-Cas9 the DNA oligonucleotides sgLMNB1-A and sgLMNB1-B (target sequence: GGGGTCGCAGTCGCCATGGC) were annealed and ligated into the BbsI sites downstream of a human U6 promoter into plasmid pU6(BbsI) chimaeric RNA. The U6-sgRNA fragment was then PCR amplified and cloned into the AscI site of CAG-Cas9-EF1-BFP. The same approach was used to clone sgRNA against LMNA (target sequence: TGGGACGGGGTCTCCATGGC) and AAVS1 (target sequence: GGGGCCACTAGGGACAGGAT) using oligonucleotide pairs sgLMNA-A/−B and sgAAVS1-A/−B. To construct the targeting vector pTetO-CREB1-3xFLAG a 1.5 kb fragment containing the CREB1 homology arms (HA) flanking 3X FLAG Tag insert was synthesized (Thermo Fischer Scientific). This fragment was then cloned via SgfI/ SpeI sites into the plasmid backbone of pTLR-donor-tetO. The same strategy was used to derive pTetO-GABPA-3xFLAG (2 kb HA), pTetO-AAVS1-3xFLAG (1.6 kb HA) and pTetO-LMNA-3xFLAG (1.1 kb HA) that were synthesized and cloned into the SgfI/SpeI sites of pTLR-donor-tetO. To clone pTetO7-LMNB1-GFP, the 448 bp SalI fragment was isolated from pTLR-donor-tetO and ligated upon end filling into the SnaBI site of AICSDP-10:LMNB1-mEGFP (Addgene #87422). All coding regions and functional elements of the constructed plasmids were confirmed by Sanger sequencing. The plasmids will be distributed via the Addgene repository (www.addgene.org).

### Generation of reporter cell lines

The human IPS cell lines BCRT and JWT (a kind gift of Harald Stachelscheid, BIH, Berlin) were used to generate TLR-Cas9 transgenic cell lines. These lines were grown in feeder-free conditions in Essential 8 (E8) or E8 Flex complete media (Thermofischer Scientific, #A1517001) on Vitronectin (Life Technologies, #A14700) coating in a 6-well cell culture plate. Plasmid DNA (pbs-U6-sgAAVS1-T2 (Addgene ID 41818), pAAVS1-TLR [11] and pAAVS1-NEOwt-CAG-Cas9v3a-bpA vector were diluted at a concentration of 0.5 μg/μl in deionized water. The AAVS1-TLR and -Cas9 Knockin vectors harbor a Puromycin and Neomycin (G418) antibiotic resistance marker respectively for the selection of targeted clones. Passaging was performed using PBS-EDTA dissociation buffer in fresh media containing Y27632 selective ROCK Inhibitor (Tebu-Bio, #21910–2301-2). The cells were transfected using Lipofectamine 3000 transfection reagent (Life Technologies, #L3000001) using 1 μg plasmid each for sgRNA, Cas9 and TLR vectors following manufacturer’s protocol. Puromycin (Sigma-Aldrich #P8833-25MG) (0.5 μg/ml) selection was started 48 h after transfection and continued until single colonies were formed. Puromycin resistant single clones were picked and then selected by using G418 (ThermoFisher #11811064) at the concentration of 100 μg/ml for 10 days. After the antibiotic selection, 24 colonies were expanded for genotyping. Genomic DNA was isolated using Wizard genomic DNA purification kit (Promega #A1125). Genotyping PCRs were performed for knock-in of TLR construct (puro 5′) and Cas9 (Neo 5′) as well as AAVS1 WT locus specific as a control. The PCR reaction for TLR Knockin was performed using primers ST_puro_gt_fw and ST_puro_gt_rv, Phusion HF DNA polymerase (NEB # M0530 L) and 200 ng genomic DNA using following conditions; 98 °C for 3 min, 35 cycles of 98 °C 30 s, 58 °C 30 s and 72 °C for 90 s. Cas9 KI was confirmed by Neo 5′ KI PCR using primers ST_neo_gt_fw and ST_neo_gt_rv using conditions at 98 °C for 5 min, followed by 35 cycles of 98 °C 30 s, 61 °C 30s, 72 °C for 90 s. AAVS1 WT PCR was performed using primers hAAVS1-For and hAAVS1-Rev by amplifying at 98 °C for 5 min, followed by 40 cycles of 98 °C 30 s, 60 °C 30 s, 72 °C for 45 s. After confirmation of TLR and Cas9 insertion by genotyping, selected clones were tested for activity of the TLR allele by FACS-based assay and one clone was chosen for further assays. The expression of Cas9 and of pluripotency markers in TLR/Cas9 reporter cells was confirmed by immunofluorescence staining.

HEK293 cells were maintained in Dulbecco’s Modified Eagle’s medium with Glutamax (Gibco) supplied with 10% fetal bovine serum (Gibco). The HEK^TLR6^ line was generated by using pAAVS1-TLR6, the same AAVS1 targeting vector as described above for the TLR construct with the difference that the TLR6 insert can be used for both plasmid and ssODN-based repair. The transfections for generation of the HEK^TLR6^ line were performed using sgRNA for AAVS1 and Cas9 plasmids (750 ng each) using Xtreme-gene transfection reagent (Roche #6366244001). Antibiotic selection was performed using 0.4 μg/ml Puromycin. Single clones were generated and genotyped in the same way as described above for the iPS TLR/Cas9 lines. All cell lines were confirmed for the absence of mycoplasma using the PCR assay of Uphoff and Drexler [39].

#### Transfection of cells

TLR/Cas9 transgenic iPS cells were passaged one day prior to transfection using PBS-EDTA dissociation buffer. Plasmid vectors sgRNA-Rosa3, TLR donor template and one or a combination of Rad18^UBD^ or RNF169^UBD^ fusion vectors (750 ng each) were transfected using Lipofectamine 3000 (Thermo Fisher Scientific) according to manufacturer’s protocol. HEK^TLR6^ cells were seeded in 24-well plates (50,000 cells per well) one day before transfection. Cells were transfected with pU6Rosa-CAG-Cas9, pTLR-donor-tetO or pTLR-donor-UAS template and one or two plasmids for the expression of Rad18^UBD^ or RNF169^UBD^ fusion proteins (375 ng each) using Xtreme gene transfection reagent (Merck) according to manufacturer’s protocol. All samples were transfected in triplicate wells. For the targeting of endogenous genes in HEK293, cells were seeded in 48-well plates (50,000 cells per well) one day before transfection. The transfection of HEK293 cells were performed using sgRNA for the targeted locus, Cas9, targeting vector and/or the fusion proteins (360 ng each plasmid per well) using Xtreme-gene transfection reagent.

#### Analysis of HDR and NHEJ by flow cytometry

FACS analysis for TLR reporter assays was performed upon 72 h after transfection. For preparation of cells for FACS analysis the medium was aspirated from wells and each well was washed with PBS. Accutase (Thermo Fisher Scientific, #A1110501) was used to detach iPS cells while Trypsin was used for the HEK cells. The cells were collected after adding appropriate media by centrifugation at 300 g for 4 min and finally resuspended in 500 μl PBS. The cells were kept on ice and FACS analysis was performed immediately. Single cells were gated for BFP positive populations and the frequency of Venus (HDR) and RFP (NHEJ) positive cells was determined using a BD LSR Fortessa flow cytometer (BD Biosciences). The analysis of the entire population (without BFP gating) leads to the same proportion of Venus and RFP positive cells but lower absolute numbers. The results from triplicate wells of each sample were used to calculate the mean value and standard deviation. For the FACS analysis of LMNB1-GFP Knockin in HEK cells, the medium was aspirated from wells and each well was washed with PBS. The cells were then harvested using Trypsin and collected down after adding appropriate media by centrifugation at 300 g for 4 min and finally resuspended in 500 μl PBS. The cells were kept on ice and FACS analysis was performed immediately. For LMNB1-GFP assay single cells were gated for the frequency of GFP positive cells reporting for HDR using BD LSR Fortessa flow cytometer (BD Biosciences). Flow cytometry settings were controlled by using untransfected cells or cells transfected with a BFP or Venus expression vector as shown in Figure S7.

#### Amplicon sequencing

For the TLR locus the frequency of NHEJ and HDR was determined by Illumina amplicon sequencing via Genewiz (Amplicon EZ; GENEWIZ Germany GmbH). For this purpose, DNA was isolated from pooled triplicates of the same experiment used for FACS (Fig. 4). An outer PCR reaction (591 bp) was performed using primers located outside the homology arms, while a second PCR reaction was performed using primers amplifying a shorter PCR product of 245 bp. For the first PCR reaction primers CAG2 and Venus-rev were used alongwith Optitaq DNA polymerase (Roboklon, # E2600–02) and 200 ng DNA per reaction using these conditions; initial denaturation at 95 °C for 1 min, followed by 35 cycles of 95 °C 20 s, 61 °C 35 s and 72 °C for 40 s and final extension at 72 °C for 5 min. For the second PCR amplification, Venus-for-Ilumina and Venus-rev-illumina Optitaq DNA polymerase and 20 ng of purified 1st PCR product following these conditions; initial denaturation at 95 °C for 2 min, followed by 35 cycles of 95 °C 20s, 61 °C 25 s, 72 °C for 40 s and final extension 72 °C for 5 min.

For the LMNA, GABPA, CREB1 and AAVS1 loci, the frequency of NHEJ and HDR was determined by Illumina amplicon sequencing via Genewiz (Amplicon EZ; GENEWIZ Germany GmbH). DNA was isolated from each well and a first PCR was performed using primers with at least one primer binding site locating outside the homology arm (Table S1). For the CREBP1 and GABPA gene, the PCR reaction was performed using hCREBP1 5HDR-F + hCREBP1-3HA intern-R and hGABPA 5HA intern-F + hGABPA 3-HR-R primers, LongAmp DNA polymerase (Neb #M0323S) and 200 ng DNA per reaction using these conditions; initial denaturation at 94 °C for 30 s, followed by 35 cycles of 94 °C 15 s, 59 °C 40 s and 65 °C for 1 min 30 s. Final extension was done at 65 °C for 10 min. For the AAVS1 and LMNA locus, the PCR reaction was performed using HR-AAVS1-F + AAVS1-R and LMNA-outer-F + LMNA-outer-R primers, LongAmp DNA polymerase (Neb #M0323S) and 200 ng DNA per reaction using these conditions; initial denaturation at 94 °C for 30 s, followed by 35 cycles of 94 °C 15 s, 61 °C 40 s and 65 °C for 1 min 30 s/2 min (AAVS1/LMNA). Final extension was done at 65 °C for 10 min. The second PCR amplification was performed using following primers (Primer LMNA 3′ inner fw_illumina, Primer LMNA 3′ inner rev_illumina, hGABPA_ intFw_illumina, hGABPA_ intRev_illumina, hCREB1_IntFw_Illumina, hCREBP1_intRev_Illumina, AAVS1 inner fw_illumina, and AAVS1 inner rev_illumina), Q5 DNA polymerase (Neb #M0491S) and 50 ng of gelextracted 1st PCR product with these conditions; initial denaturation at 98 °C for 30 s, followed by 35 cycles of 98 °C 10 s, 62 °C 30 s, 72 °C for 15 s and final extension at 72 °C for 2 min. The PCR products were purified on column using the GeneJET PCR Purification Kit (Thermo Scientific, #K0702). 2 μg of each purified PCR reaction was submitted for deep sequencing. The analysis of deep sequencing data was performed using Crispresso [40] and Crispresso2 (http://crispresso.pinellolab.partners.org/) online bioinformatics tools designed for analysis of Crispr-based gene editing data. Additionally, a custom Amplicon-sequencing analysis pipeline (licensed from Bioinformatics. Expert UG, Berlin, Germany) was used for data analysis which needs the rawdata fastq-files for the paired reads as well as the reference sequence provided as fasta-file. The main functionalities of that pipeline were implemented in R statistics (version: 3.6.0). The alignment algorithm was performed using the R function “pairwiseAlignment()” from the R package Biostrings (version: 2.52.0). In a first step, the paired reads were aggregated and cleaned to reduce potential technical sequencing errors. This was done for each pair of reads as following: First, the R2-read was converted to its reverse complement sequence, the quality score was reversed. Second, the R1- and R2-read were aligned locally to identify the overlap between the read pairs. The local alignment was performed with very high penalty values for gap opening and gap extension to avoid an alignment with gaps, which might shift the bases during aggregation. Third, R1 and the reverse complement R2-sequence (rcR2) were stitched together at the aligned sequence. If bases differ between R1 and rcR2, the base was chosen, which had the highest quality score on that position. Missing bases at the end were filled with “N”. Fourth, the 15 bases from the 5′ and 3′ ends of the reference sequence were aligned locally to the aggregated sequence to identify the sequence boundaries. In this step, two mismatches or two Insertions/Deletions were allowed. Afterwards, all bases in front or after the alignment were chopped. So, a final aggregated sequence was created for each read, which starts and ends with the boundary bases of the reference sequence. In the second part of the pipeline, all aggregated reads were then aligned globally to the reference sequence (alignment reward/penalty scores: match = 1, mismatch = 0, gapOpen = − 2, gapExtension = 0). The alignment scores were chosen to favor long gaps over small gaps and mismatches. Finally, all unique aggregated sequences were sorted and counted.

### Statistics

For all experiments, data are shown as mean ± standard deviation. Statistical significance was determined as indicated in each results part. A *P* value of less than 0.05 was considered statistically significant. Statistical analysis was performed using GraphPad Prism 8 (GraphPad Software Inc., San Diego, USA).

### Synthetic Oligodeoxynucleotides

Synthetic oligonucleotides used in this study were purchased from Eurofins Genomics (Ebersberg, Germany) and are shown in Table S1.

## Supplementary information

**Additional file 1: Figure S1.** *Analysis of Venus positive cells in HEK*^*TLR6*^ *cells upon transfection with Cas9 and Rosa26 specific sgRNA.* Positive cells (0.48%) were isolated by FACS sorting and the target region was amplified from genomic DNA, subcloned and sequenced. Two analysed clones (Seq clone A, B) showed a deletion of 14 bp that removes a part of the *Rosa26* target sequence and restores the Venus reading frame and the critical Arginine codon 96 (R96). The adjacent codons 95 and 97 are also replaced (T, W) but seemingly do not disrupt Venus fluorescence. (PDF, 195 kb) (EPS 561 kb)

**Additional file 2: Figure S2.** *Intracellular localization of Rad18UBD protein in HEK cells.* HEK293 cells were transfected with an expression vector for FLAG tagged Cas9 (pX330, Addgene 42,230) (A) or FLAG tagged Rad18UBD (B) using XtremeGene transfection reagent. After 48 h cells were treated for 10 min with H_2_O_2_ (500 μM) and fixed after 1 h in 4% paraformaldehyde. Fixed cells were stained in PBS, 0.2% Triton X-100, 3% BSA with antibodies against phospho-H2AX (mouse mAb, clone JBW301, Millipore #05–636, 1:500) and FLAG Tag (rabbit mAb, Cell Signaling Technology # 14793, 1:800) for 1 h. After washing slides were incubated for 1 h with secondary goat antibodies against mouse IgG (Alexa Fluor 594, Life Technologies #A-11032, 1:1000) and rabbit IgG (Alexa Fluor 488, Life Technologies A11034, 1:1000), washed and incubated for 10 min in Hoechst 33342 stain (Life Technologies H3670, 1:2000). After washing images were acquired using a Keyence BZ9000 microscope. In (B) the FLAG Tag signals are colocalized with γH2AX foci. (PDF, 986 kb)

**Additional file 3: Figure S3.** *DSB repair modification by Gal4-Rad18*^*UBD*^ *and -RNF169*^*UBD*^ *fusion proteins in HEK*^*TLR6*^ *reporter cells.* Fusion constructs for Gal4 or BRCA1 with Rad18^UBD^ or RNF169 ^UBD^ were cotransfected with the matching TLR HDR repair template (TLR-donor-UAS), sgRNA and Cas9 into HEK^TLR6^ cells. The frequency of Venus and RFP positive cells was measured by FACS analysis 72 h after transfection. The HDR frequency is reported by Venus (green bars) while the fraction of NHEJ events in reading frame + 2 is reported by RFP expression (red bars). The bars represent mean values ± standard deviation, Y-axis represents the frequency of Venus or RFP positive cells in percent while the X-axis shows samples transfected in combinations, as shown in the table below. Samples 1 and 2 are controls showing the basic frequency of RFP^+^ only and of Venus^+^ cells in addition when TLR-donor-UAS is provided as repair template. As compared to BRCA1 alone (sample 3) the expression of BRCA1-Rad18^UBD^ or RNF169^UBD^ fusions strongly increased the Venus/RFP ratio to values of 2.94 and 3.0 (samples 4 and 5). The expression of Gal4-Rad18^UBD^ or Gal4-RNF169^UBD^ increased the Venus/RFP ratio by a factor of 4 or 5.9, from 0.34 (sample 2) to values of 1.37 or 2.0 (samples 6 and 7). The combined expression of Gal4-UBD with BRCA1-UBD fusions further increased the Venus/RFP ratio to a value of 3.14 in fusion with Rad18^UBD^ and to 3.29 in fusion with RNF169^UBD^. This increase however was mostly if not entirely attributed to the effect of BRCA1-UBD fusion proteins alone that lead to HDR/NHEJ ratios of 2.94 and 3.0 (samples 4 and 5). Data from three independent experiments, each with three replicates per sample, are presented as mean values ± S.D. Significance of samples in comparison to the control sample 2 with sgRosa/Cas9 and TLR-donor-UAS was determined by two-way ANOVA and Dunnett’s multiple comparison tests with ***P < 0.001 (HDR) and ###P < 0.001 (NHEJ). (PDF, 271 kb). Raw data are shown in the Supplementary data file. (EPS 313 kb)

**Additional file 4: Figure S4.** *Sequence-based DSB repair assay using Rad18*^*UBD*^ *and RNF169*^*UBD*^ *fusion proteins in HEK*^*TLR6*^ *reporter cells.* Transfected reporter cells from one of the assays analysed by FACS (Fig. 4; experiment 1 in the supplement data file) were used for PCR amplification of the reporter target region from genomic DNA (A), isolated from pooled cells of the triplicate samples used for FACS analysis 72 h after transfection. (B) PCR products were sequenced by amplicon sequencing and the fraction of reads showing HDR (green bars) or Indel events (red bars) is shown in relation to the total number of reads with gene editing events on the Y-axis and was used to calculate the ratio of HDR/NHEJ DSB repair. The X-axis shows the transfected samples and the selection of cotransfected plasmids below. Samples 1 and 2 are controls showing the basic frequency of Venus^+^ and RFP^+^ cells upon transfection with Cas9 and sgRNA or in combination with TLR-donor-tetO as repair template. The fraction of sequence reads representing the 14 bp deletion causing Venus background expression (Figure S1) is given as ‘percent background’. Raw data are shown in the Supplementary data file. (EPS 1295 kb)

**Additional file 5: Figure S5.** *Distribution of reading frames within the mutagenic NHEJ repair products in HEK*^*TLR6*^ *reporter cells.* Using CRISPResso analysis of the amplicon sequencing data shown in Figure S4 we calculated for each sample the distribution of the reading frames + 1 (Venus expression frame), + 2 and + 3 among the repair products showing + 1 insertions or deletions from − 1 to − 12 nucleotides. RFP expression becomes activated in the TLR-6 construct in the reading frame + 2 by the deletion of 1, 4, 7 or 10 nucleotides. Of note, the frequency of reading frame + 2 products is lowest in sample 1 in the absence of pTLR-donor. Raw data are shown in the Supplementary data file.

**Additional file 6: Figure S6.** *DSB repair modification by Rad18*^*UBD*^ *and RNF169*^*UBD*^ *fusion proteins in hiPS reporter cells.* Fusion constructs for BRCA1 or TetR with Rad18^UBD^ or RNF169 ^UBD^ were cotransfected with the TLR HDR repair template (TLR-donor-tetO), sgRNA and Cas9 and analysed for Venus and RFP positive cells using FACS analysis 72 h after transfection. The HDR frequency is reported by Venus (green bars) while the fraction of NHEJ events in reading frame + 3 is reported by RFP expression (red bars). The bars represent mean values ± standard deviation, Y-axis represents the frequency of Venus or RFP positive cells in percent while the X-axis shows samples transfected in combinations, as shown in the table below. Controls show the basic frequency of RFP^+^ cells upon transfection of Cas9 plus sgRNA, and of Venus^+^ and RFP^+^ cells in the presence of the repair template TLR-donor-tetO. Expression of i53, BRCA1 or BRCA1-UBD fusion proteins showed levels of Venus^+^ cells that were statistically not significantly different as compared to sample 2, except for BRCA1-RNF169^UBD^ (samples 5–8) and the TetR-UBD fusions (sample 7 and 8). The coexpression of i53 with a single Rad18^UBD^ or RNF169 ^UBD^ fusion (samples 9–12) significantly enhanced the Venus/RFP ratio by a factor of 7–16.6. The combined expression of the two fusion proteins BRCA1-Rad18^UBD^ and TetR-Rad18^UBD^ (sample 13) or BRCA1-RNF169^UBD^ and TetR-RNF169^UBD^ (sample 14) led to 4–6-fold increase of the Venus/RFP ratio . The expression of full length Rad18 or RNF169 proteins (sample 15 and 16) did not show significant difference to the control sample. Data are shown as mean values ± SD from two independent experiments into BCRT or JWT iPS cells, each with three replicates per samples, normalized to the values of sample 2 as control. Statistical significance of values in comparison to the control sample 2 with sgRosa/Cas9 and TLR-donor-tetO was determined by two-way ANOVA and Dunnett’s multiple comparison tests with *P < 0.05, **P < 0.01, ***P < 0.001 (HDR) and ##P < 0.01, ###P < 0.001 (NHEJ). Raw data are shown in the Supplementary data file. (EPS 449 kb)

**Additional file 7: Figure S7.** Flow cytometry controls for DSB repair assay in TLR reporter cells. FACS gating scheme for BFP and Venus positive cells in HEK^TLR6^ reporter cells. Cells were untransfected (A) or transfected either with pU6Rosa-CAG-Cas9 with pTLR donor (B) or with a Venus (C) or BFP (D) expression plasmid. Single cells were gated by using a forward scatter plot. Transfected cells were gated based on expression of BFP, Venus or RFP compared to the non-transfected control. The fraction of positive cells in the defined windows is indicated. Raw data are shown in the Supplementary data file. (EPS 1761 kb)

**Additional file 8: Table S1.** List of primers and oligonucleotides.

**Additional file 9: Supplementary data file.** Raw data points as used for Figs. 3, 4, 5, 6, and Fig. S3, S4, S5, S6, S7.

## Data Availability

All data generated and analysed in this work are available in this published article and the Supplementary data file. Plasmids constructed in this study are available from Addgene (www.addgene.org).

## Abbreviations

CRISPR: Clustered regularly interspaced short palindromic repeat
Cas9: CRISPR associated protein 9
DSB: Double-strand break
FACS: Fluorescense activated cell sorting
HDR: homology directed repair
NHEJ: Non-homologous end joining
sgRNA: single guide RNA
UBD: Ubiquitin binding domain
