# Supplementary figures and images for: Enhancement of CRISPR-Cas9 induced precise gene editing by targeting histone H2A-K15 ubiquitination
Source: BMC Biotechnol. 2020 Oct 23;20:57. doi: 10.1186/s12896-020-00650-x (PMC7585302; doi:10.1186/s12896-020-00650-x)

**A****FLAG**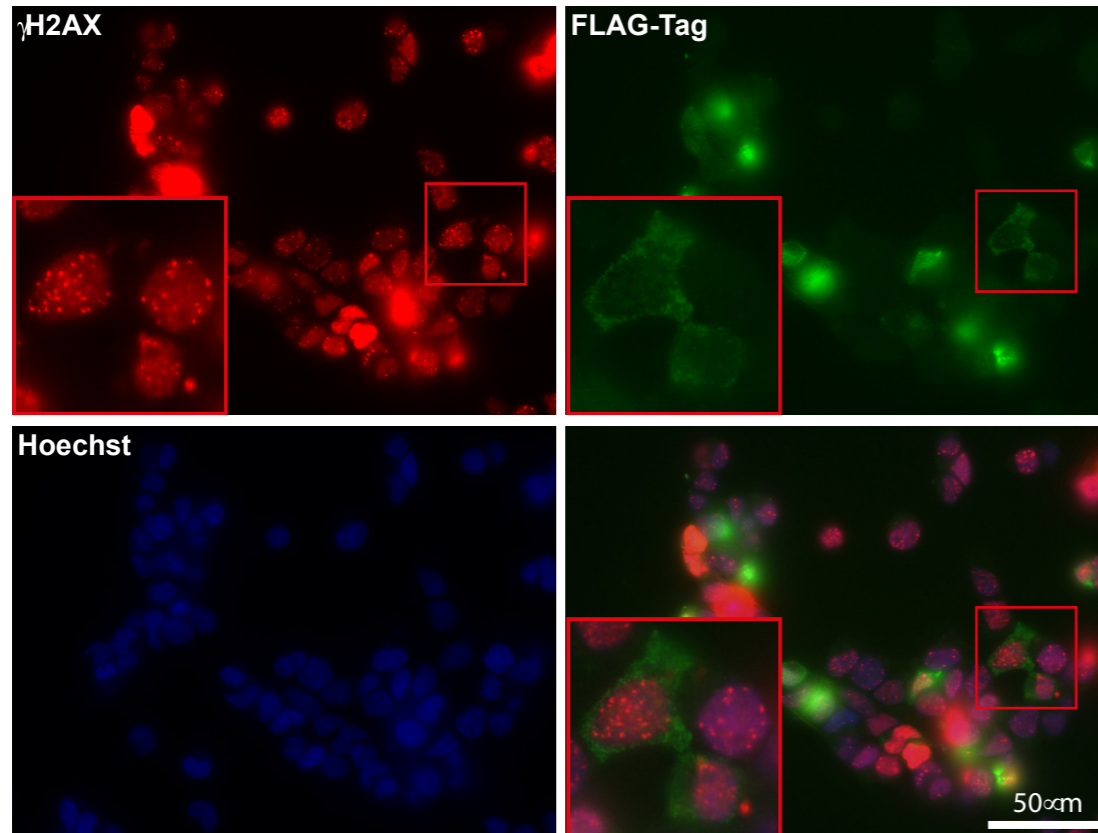**B****FLAG-Rad18UBD**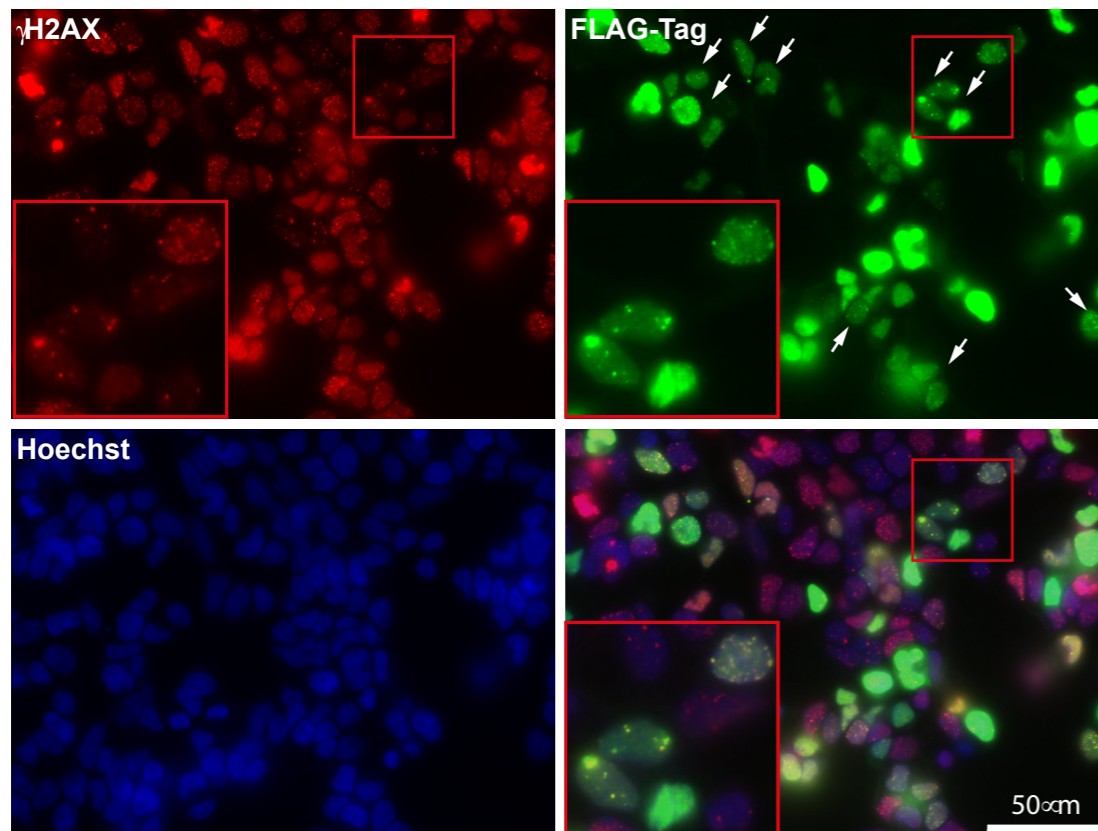

Supplement: Supplementary file 2 — Additional file 2: Figure S2. Intracellular localization of Rad18UBD protein in HEK cells. HEK293 cells were transfected with an expression vector for FLAG tagged Cas9 (pX330, Addgene 42,230) (A) or FLAG tagged Rad18UBD (B) using XtremeGene transfection reagent. After 48 h cells were treated for 10 min with H2O2 (500 μM) and fixed after 1 h in 4% paraformaldehyde. Fixed cells were stained in PBS, 0.2% Triton X-100, 3% BSA with antibodies against phospho-H2AX (mouse mAb, clone JBW301, Millipore #05–636, 1:500) and FLAG Tag (rabbit mAb, Cell Signaling Technology # 14793, 1:800) for 1 h. After washing slides were incubated for 1 h with secondary goat antibodies against mouse IgG (Alexa Fluor 594, Life Technologies #A-11032, 1:1000) and rabbit IgG (Alexa Fluor 488, Life Technologies A11034, 1:1000), washed and incubated for 10 min in Hoechst 33342 stain (Life Technologies H3670, 1:2000). After washing images were acquired using a Keyence BZ9000 microscope. In (B) the FLAG Tag signals are colocalized with γH2AX foci. (PDF, 986 kb) [file 12896_2020_650_MOESM2_ESM.pdf]

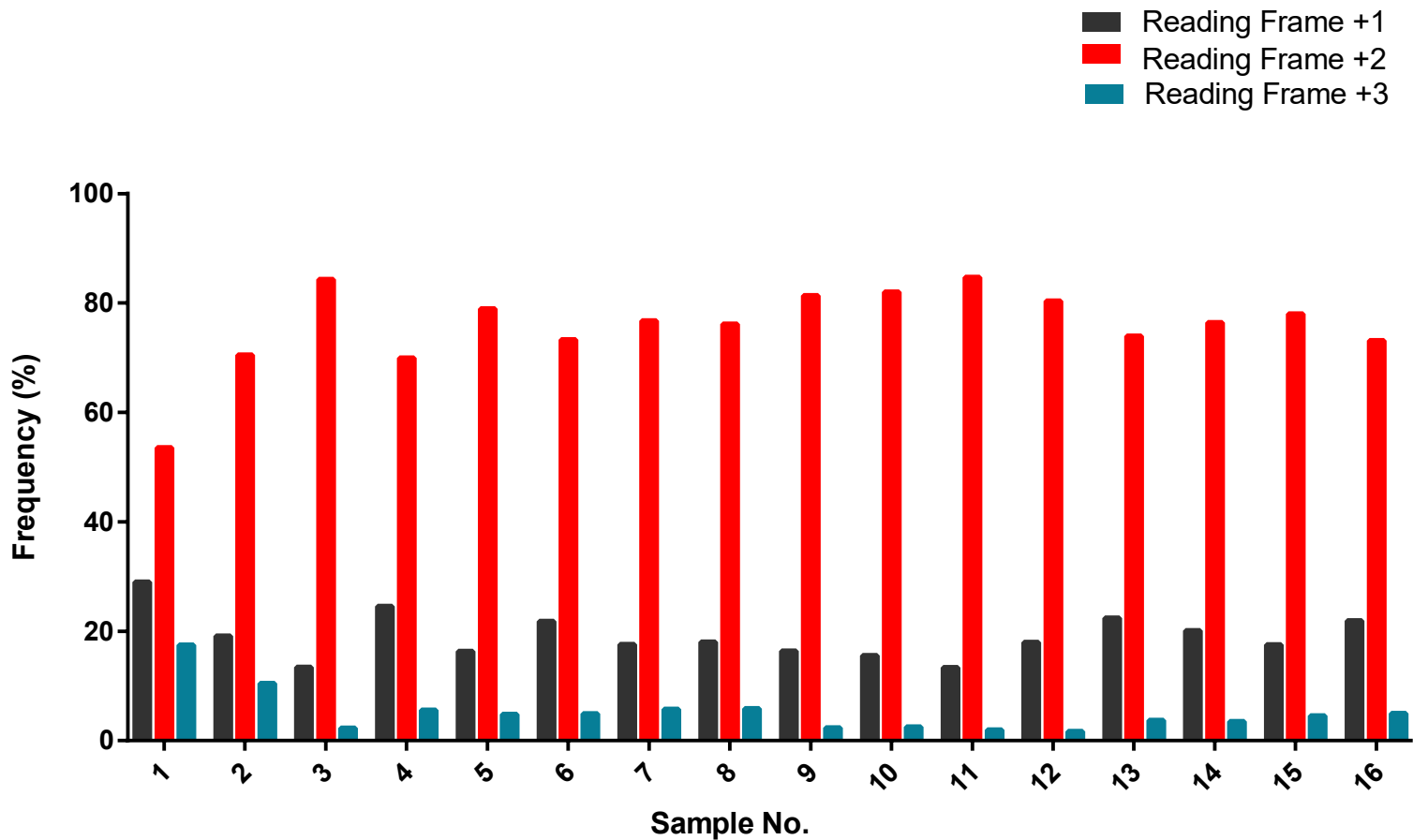

Supplement: Supplementary file 5 — Additional file 5: Figure S5. Distribution of reading frames within the mutagenic NHEJ repair products in HEKTLR6 reporter cells. Using CRISPResso analysis of the amplicon sequencing data shown in Figure S4 we calculated for each sample the distribution of the reading frames + 1 (Venus expression frame), + 2 and + 3 among the repair products showing + 1 insertions or deletions from − 1 to − 12 nucleotides. RFP expression becomes activated in the TLR-6 construct in the reading frame + 2 by the deletion of 1, 4, 7 or 10 nucleotides. Of note, the frequency of reading frame + 2 products is lowest in sample 1 in the absence of pTLR-donor. Raw data are shown in the Supplementary data file. [file 12896_2020_650_MOESM5_ESM.pdf]
