# Supplementary material for: Enhancement of CRISPR-Cas9 induced precise gene editing by targeting histone H2A-K15 ubiquitination
Source: BMC Biotechnol. 2020 Oct 23;20:57. doi: 10.1186/s12896-020-00650-x (PMC7585302; doi:10.1186/s12896-020-00650-x)
Supplement: Supplementary file 8 — Additional file 8: Table S1. List of primers and oligonucleotides. [file 12896_2020_650_MOESM8_ESM.pdf]

Table S1: Primers and Oligonucleotides used in this work

Name	Sequence (5´- 3´)
TLRvenus-1	ATCCTTAATTAAGCCGCCACCATGGTGAGCAAGGGCGAGGAGCTG
TLR6-1	CCATCTTCTAGAAAGACTGGAGTCTGGACGTAGCCTTCGGGCATG
TLR6-2	GACTCCAGTCTTTCTAGAAGATGGATCTTCTTCAAGGACGACGGC
TagRFP-2	ATTTACGCGTGCGATCGCTCAATTAAGTTTGTGCCCCAGTTTG
TLRtv-1	GTGAGCAAGGGCGAGGAGCTG
TLRtv2	GGTGGCGGCTTAATCAAGCTTATC
sgRosa-A	CACCGACTCCAGTCTTTCTAGAAGA
sgRosa-B	AAACTCTTCTAGAAAGACTGGAGTC
UAS-for	TCGACCGCAAGCTTGCATGCCTGCAGGT
UAS-rev	TCGACATGCGCTAGCGCTAGAGTCTCCG
Gal4-for	TCCTTAATTAATAATAATACGACTCACTATAGGGGCCACCATGAAGCTACTGTCTTCTATCGAAC
Gal4-rev	ACGACGATCGGCTACCTCCTCCTCCGCTTCCTCCTCCTCCGCTTCCACCTCCTCCGTGCACCTTCCTCTTTTTCTTAGGCGATACAGTCAACTGTCTTTGACC
Rad18-for	TCCTTAATTAATAATACGACTCACTATAGGGGCCACCATGGACTACAAGGATGACGACGATAAACCCTCGACATCCACTTTGAAACAAG
Rad18-rev	CATTTACGCGTCTACCTTTTGTGAACAGAACTTCTGAGG
Rad18-for2	TCCTTAATTAATAATACGACTCACTATAGGGGCCACCATGGACTCCCTGGCCGAGTCT
Rad18-rev2	CATTTACGCGTTTAATTCCTATTACGCTTGTTTCTTGGT
RNF169-for2	TCCTTAATTAATAATACGACTCACTATAGGGGCCACCATGGACTACAAGGATGACGACGATAAAAAGCTTCAGCAAGAGGAAGAAGACC
RNF169-rev	CATTTACGCGTCTACTTGGCCCCGGCCATGTTG
RNF169-for	TCCTTAATTAATAATACGACTCACTATAGGGGCCACCATGGCGGCTGCAGGTCCGAGTA
BRCA1-for	TCCTTAATTAATAATACGACTCACTATAGGGGCCACCATGGATTTATCTGCTCTTCGCGTTG
BRCA1-rev	CATTTACGCGTTCAGTAGTGGCTGTGGGGGATCT
BRCA1-rev2	ATCGCGATCGGCTACCTCCTCCTCCGCTTCCTCCTCCTCCGCTTCCACCTCCTCCGTAGTGGCTGTGGGGGATCTGG
I53-for	TCCTTAATTAATAATACGACTCACTATAGGGGCCACCATGTTGATTTTCGTGAAAACCCTTAC
I53-rev	CATTTACGCGTTCAACGAAGTCTCAACAGAGGATG
TetRsc4-for	ATCGATTAATTAATAATAATACGACTCACTATAGGGGCC
TetRsc4-rev	tacgaCGatcgGCTACCTCCTCCTCCGCTTCCTCCTCCTCCGCTTCCACCTCCTCCGTGCACCTTCCTCTTTTTCTTAGGAGAG
TetR-for	ATTAATAATACGACTCACTATAGGGGCCACCATGTCCAGACTGGACA
TetR-rev	TCATTTACGCGTTCACACGTGCTTTCTCTTTTTGGG
scTetR-for2	CTGAAATGTGAGTCTGGCTCTCACGTGGGAGGAGGTG
scTetR-rev2	CTGCAATCATTTACGCGTTCACACGTGCACCTTCCTCTTTTTC
5HA HR Rev	CGGGATTCTCCTCCACGTCACCGCA
hAAVS1 For	TCCAGGGGTCCGAGAGCTCAGCTAG
ST_puro_gt_fw	TCCCCTCTTCCGATGTTGAG
ST_puro_gt_rv	GCGGTCCGGATCGACG
ST_neo_gt_fw	CTTACCATCCCTCCCTCGACT
ST_neo_gt_rv	GAACCTGCGTGCAATCCA
Venus TLR fw	CACCATGGTGAGCAAGGGCGAG
TagRFP1rev	GGTACAGCATCTCGGTGTTGG
Primer CAG2	TTCGGCTTCTGGCGTGTGACC
Primer Venus-rev	CGTTCTTCTGCTTGTCGGCGG
Primer Venus-for-*Ilumina adapter*	*ACACTCTTTCCCTACACGACGCTCTTCCGATCT*TCAAGTCCGCCATGCCCGAAG
Primer Venus-rev-*Ilumina*	*GACTGGAGTTCAGACGTGTGCTCTTCCGATCT*CGTTCTTCTGCTTGTCGGCGG
Primer LMNA 5' outer fw	CACTCCTGGCACACTGAGATG

Primer LMNA 3' outer rev	GGTGCCCAGAGTTCTTCGAG
Primer LMNA 3' inner fw_illumina	*ACACTCTTTCCCTACACGACGCTCTTCCGATCT*ACTCCGAGCAGTCTCTGTCC
Primer LMNA 3' inner rev_*illumina*	*GACTGGAGTTCAGACGTGTGCTCTTCCGATCT*ACCGCCAAGCGATCATTGAG
sgLMNA-A	CACCGTGGGACGGGGTCTCCATGGC
FunsgLMNA-B	AAACGCCATGGAGACCCCGTCCCAC
Primer hGABPA 5HA intern-F	CTCTTATTGGATACAGTGCAGC
Primer hGABPA 3' HR rev	AGCTATCTGCCAGTCTCATG
Primer hGABPA_ intFw_*illumina*	*ACACTCTTTCCCTACACGACGCTCTTCCGATCT*AATTGCCCAGCCAGTCACAG
Primer hGABPA_ intRev_*illumina*	*GACTGGAGTTCAGACGTGTGCTCTTCCGATCTATGAACCTGCCTTCACACTGC*
Primer hCREB1 5' HDR-F	TGCCAAATCCAATGTCAGAGAG
Primer hCREB1-3HA intern-R	CAGGTGTTGGAACATTCACAG
Primer hCreb_intFw_*illumina*	*ACACTCTTTCCCTACACGACGCTCTTCCGATCT*GCTTGTAGGGAAGCAGCTCG
Primer hCreb_intRev _*illumina*	*GACTGGAGTTCAGACGTGTGCTCTTCCGATCT*CAGGTGTTGGAACATTCACAG
Primer HR_AAVS1-F	CTGCCGTCTCTCTCCTGA GT
Primer AAVS1-R	CCAGCTCCCCTGCTTCTTGGCCAC
Primer AAVS1 inner fw_*illumina*	*ACACTCTTTCCCTACACGACGCTCTTCCGATCT*CGGTTAATGTGGCTCTGGTT CTG
Primer AAVS1 inner rev_*illumina*	*GACTGGAGTTCAGACGTGTGCTCTTCCGATCT*CAATATCAGGAGACTAGGAAG GAGG
sgAAVS1-A	CACCGGGGGCCACTAGGGACAGGAT
sgAAVS1-B	AAACATCCTGTCCCTAGTGGCCCCCG
sgLMNB1-A	CACCGGGGGTCGCAGTCGCCATGGC
sgLMNB1-B	AAACGCCATGGCGACTGCGACCCCC
